# Health systems research in fragile settings 

**DOI:** 10.2471/BLT.19.233965

**Published:** 2019-06-01

**Authors:** Alastair Ager, Shadi Saleh, Haja Wurie, Sophie Witter

**Affiliations:** a NIHR Research Unit on Health in Situations of Fragility, Queen Margaret University, Queen Margaret Drive, Edinburgh, EH21 6UU, Scotland.; b NIHR Research Unit on Health in Situations of Fragility, Global Health Institute, American University of Beirut, Beirut, Lebanon.; c NIHR Research Unit on Health in Situations of Fragility, College of Medicine and Allied Health Sciences, University of Sierra Leone, Freetown, Sierra Leone.

Population health indicators have improved in recent decades. Deaths in children younger than five years have declined from over 16 million in 1970 to around 5 million in 2016^1^ and life expectancy at birth has increased from 58 to over 70 years in the same period.^2^

However, a major constraint to such progress, and in some contexts a potential source of reversal, is fragility. Of the 10 countries with the highest rates of infant mortality, seven are classified as fragile states. Of the 20 countries with the weakest progress on reducing maternal mortality from 1990 to 2015, 14 were fragile.^3^ However, fragility is increasingly recognized as a phenomenon that is not limited to countries that meet the profile of fragile and conflict-affected states.^4^ Of those countries that currently meet the Organisation for Economic Cooperation and Development criteria of experiencing significant fragility, comprising political, societal, economic, environmental and security dimensions of instability, almost half are middle-income countries.^5^

A better understanding of the implications of health-care provision in contexts of fragility is necessary. We have, therefore, established a research unit on health in situations of fragility at Queen Margaret University, Edinburgh, Scotland. This unit is supported by the National Institute for Health Research and builds on the experiences of several institutions in post-conflict health reconstruction strategy, recovery from the Ebola virus disease outbreak in West Africa and response to political instability in the Eastern Mediterranean Region. In our analysis of how the concept of fragility is used in the global health literature, we found that fragility is most often used to describe the circumstances of states or their public health systems; however, it also increasingly addresses the relationship with communities. Where the state’s agenda and communities’ needs are poorly aligned, the strained or ruptured relationship between the two has direct implications for health. 

Understanding the weaknesses of health systems and how systems strengthening strategies may address these weaknesses must remain a core component of any approach to secure improvements in population health. However, in contexts of fragility, a key focus is needed on threats to the interface between public health provision and community processes. This exercise inevitably requires a systems for health approach^6^ that sees community, civil society, private sector actors and the state as key agents within a complex system adjusting to the prevailing drivers of fragility.

Earlier work on health systems resilience in contexts of fragility^7^^,^^8^ repeatedly pointed to the importance of this interface with communities. We are now exploring this further in three countries: El Salvador, Lebanon and Sierra Leone. In each setting, our focus is on the prevention and treatment of noncommunicable diseases and mental health and psychosocial support. Providing a response to these health needs requires an effective connection over time between diverse service providers, patients, carers and communities.

Scoping reviews in each of these fragile settings have identified recurrent challenges at this interface. When fragile settings experience acute shocks, there is a risk that the surge of local provision supported by international agencies will not strengthen health systems in the long-term. Lack of knowledge of available services, uncertain or restricted access, financial barriers or perceptions of health-care settings not constituting a safe place are also repeatedly identified across fragile settings at the community-service interface.^9^

Participatory group model building^10^ is a promising method for exploring the connections between the various actors of the systems for health in these fragile settings, and for identifying potential strategies to make these actors’ engagement more effective. Policy-level and health systems interventions are clearly relevant, but it is at the interface of public health provision and community processes that major barriers persist.

Mapping of social connection and trust^11^ can also clarify key processes supporting or inhibiting engagement within and between communities and health services in contexts of fragility.

We plan to develop a series of studies of strategic interventions designed to secure high-quality and accessible service provision in contexts of fragility. Therefore, we encourage other researchers to engage in this framing of strategic health interventions in such settings. The core goal must be achieving forms of service design and community engagement that prove durable and effective in circumstances of fragility. To the extent that these strategies are effective in building trust and social connection between (and within) the state and local communities, they may also prove of value in addressing the drivers of fragility itself.^12^
